# Hydroxyurea for secondary stroke prevention in children with sickle cell anaemia: a systematic review of clinical evidence and outcomes

**DOI:** 10.1097/MS9.0000000000001660

**Published:** 2024-01-03

**Authors:** Nicholas Aderinto, Gbolahan Olatunji, Emmanuel Kokori, Muili Abdulbasit

**Affiliations:** a Department of Medicine and Surgery, Ladoke Akintola University of Technology, Ogbomoso, Asiwaju Bola Ahmed Tinubu, Nigeria; b Department of Medicine and Surgery, University of Ilorin, Ilorin, Nigeria

**Keywords:** hydroxyurea, sickle cell anaemia, stroke

## Abstract

**Background:**

Stroke remains one of the leading complications of sickle cell anaemia (SCA) in children. Traditionally, SCA treatment focused on symptom relief. However, the high incidence of strokes in children has prompted a reevaluation of treatment, particularly hydroxyurea, for secondary stroke prevention. This study assesses hydroxyurea’s effectiveness and safety in preventing secondary strokes in paediatric SCA patients.

**Methods:**

This systematic review followed a pre-defined protocol registered with PROSPERO. Comprehensive searches were conducted across PubMed, Embase, Scopus, MEDLINE, Google Scholar, and the Cochrane Library up to August 2023. Studies were included involving paediatric SCA patients at risk of secondary stroke, assessing hydroxyurea as the primary intervention.

**Results:**

A total of six studies meeting inclusion criteria were included. The effectiveness of hydroxyurea in preventing secondary strokes, with variable responses reported across studies. Adverse effects, including mild neutropenia, are associated with hydroxyurea treatment but with variability in reported toxicity levels.

**Conclusion:**

Hydroxyurea holds promise in preventing recurrent strokes in children with SCA, though its efficacy and safety profiles vary among individuals. Optimal dosages and treatment durations require further investigation, necessitating vigilant monitoring of haematological parameters. Future research should refine dosing strategies, consider individual patient characteristics, assess long-term effects, and explore ancillary benefits beyond stroke prevention.

## Introduction

Highlights

- This systematic review underscores the effectiveness of hydroxyurea in preventing secondary strokes among children with sickle cell anaemia. The analysis of clinical evidence indicates that hydroxyurea plays a pivotal role in reducing the risk of recurrent strokes.
- Optimal dosages and treatment durations require further investigation, necessitating vigilant monitoring of haematological parameters.
- Adverse effects, including mild neutropenia, are associated with hydroxyurea treatment but with variability in reported toxicity levels.

Sickle cell anaemia (SCA) is a hereditary hemoglobinopathy that primarily affects individuals of African, Mediterranean, Middle Eastern, and Indian descent^1^. It is characterised by a genetic mutation that produces abnormal haemoglobin, haemoglobin S (HbS)^2^. In its deoxygenated state, HbS causes red blood cells to deform into a characteristic “sickle” shape, leading to impaired blood flow, tissue damage, and a wide range of clinical complications^2^. Among the myriad challenges faced by individuals living with SCA, stroke stands out as one of the most devastating and life-altering^3^. Children with SCA are at a heightened risk of experiencing overt and silent (asymptomatic) strokes^4^. Overt strokes result in observable neurological deficits, while silent strokes, often detected through neuroimaging studies, can lead to subtle cognitive impairments^5^. These neurological events can profoundly impact a child’s development and quality of life^5^.

Historically, the management of SCA has primarily focused on symptom relief and the prevention of acute complications, such as painful vaso-occlusive crises and acute chest syndrome^6^. However, recognising the high prevalence of strokes in paediatric SCA patients has prompted reevaluating therapeutic strategies. Hydroxyurea, a medication used for several decades in adults with SCA, has shown promise in modifying the disease’s clinical course^7^. It works by increasing the production of foetal haemoglobin (HbF), which interferes with the polymerisation of HbS, reducing the formation of sickle-shaped cells^7^. This mechanism of action has led to improved outcomes in adults, including decreased frequency of painful crises and hospitalisations^7^.

In recent years, researchers and clinicians have highlighted the potential benefits of hydroxyurea in preventing secondary strokes in children with SCA^8^. While it is clear that hydroxyurea can reduce the risk of vaso-occlusive events, its role in preventing strokes, particularly in the paediatric population, remains an area of active investigation^9^. This systematic review aims to assess the clinical evidence surrounding the use of hydroxyurea for secondary stroke prevention in paediatric patients with SCA. Given the unique challenges and consequences of stroke in this age group, understanding the effectiveness and safety of hydroxyurea in this context is crucial for optimising the management of SCA and improving the long-term outcomes and quality of life for affected children and their families.

## Methodology

### Study protocol and registration

This systematic review followed the protocol registered with PROSPERO (International Prospective Register of Systematic Reviews) under the registration number^10^. The protocol outlined the research question, objectives, inclusion and exclusion criteria, and the methodological approach for this systematic review. This work has also been reported in line with AMSTAR (Assessing the methodological quality of systematic reviews) Guidelines.

### Search strategy

**Figure 1:**
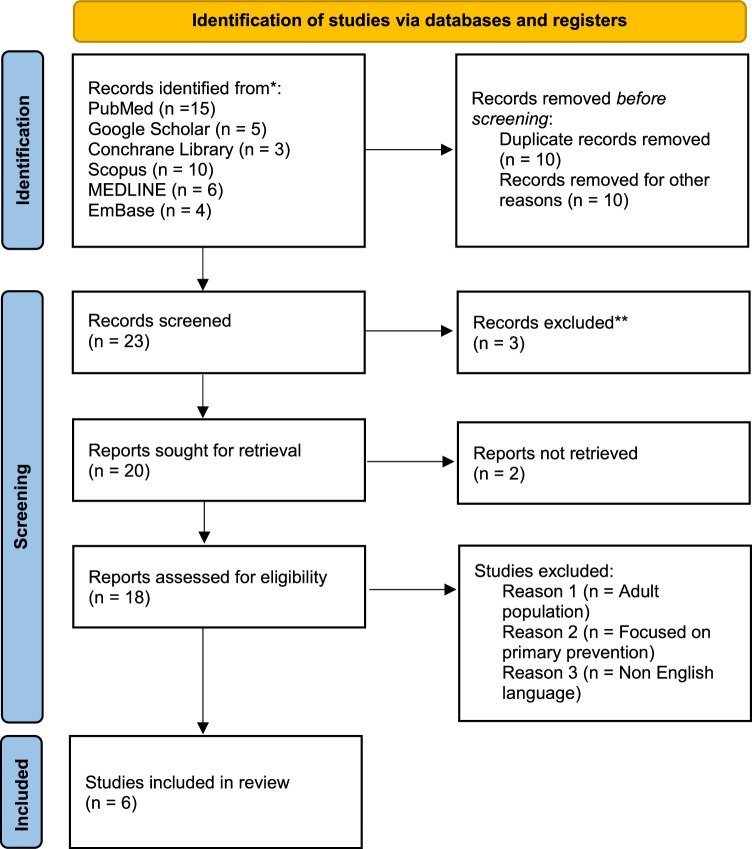
Screening process.

We conducted a comprehensive search to identify relevant studies. The search was performed in various electronic databases, including PubMed, Embase, Scopus, MEDLINE, Google Scholar, and the Cochrane Library. For PubMed, Scopus, Google Scholar and MEDLINE, search included “Sickle Cell Anaemia,” “Stroke,” and “Child.” The Boolean operators “OR” are used to broaden the search by including synonyms, and “AND” is used to narrow down the results by combining different concepts. In Embase, the search terms included controlled vocabulary (exp) terms where applicable and uses the Boolean operators “AND” and “OR” to connect different aspects of the research question. In the Cochrane Library, the search included terms related to sickle cell anaemia, stroke, prevention, and children, connected by the Boolean operators “AND” and “OR.” The search was conducted from the inception of each database until August 2023. Language restrictions to papers published in English were applied. The detailed search strategy is provided in Figure 1.

### Study selection

Per the pre-defined inclusion and exclusion criteria outlined in the PROSPERO-registered protocol, two independent reviewers (N.A. and G.O.) screened the titles and abstracts of all identified records to determine eligibility. Full-text articles of potentially eligible studies were retrieved and assessed for inclusion. Any reviewer discrepancies were resolved through discussion or involving a third reviewer (E.K.).

We included randomised controlled trials (RCTs) and observational studies, including cohort and case-control studies. Population: our focus was on studies involving paediatric populations, specifically children aged 18 or below diagnosed with sickle cell anaemia and who are at risk of experiencing a secondary stroke. Intervention: we prioritised including studies that assessed hydroxyurea as the primary intervention for preventing secondary stroke in this paediatric population. Outcome measures: to ensure a thorough evaluation of the effectiveness and impact of hydroxyurea, we sought studies that reported outcomes encompassing stroke incidence, quality of life, adverse effects related to the intervention, and patient compliance.

We limited our selection to studies published in peer-reviewed journals. This decision was made to ensure the reliability and validity of the research findings we incorporated into our systematic review. Also, we excluded papers not published in English.

### Data extractionh

Two independent reviewers systematically extracted data from the included studies using a standardised data extraction form. Information collected included study title and authors, study design, number of participants, age range, hydroxyurea dosage, treatment duration, control group, main findings, and adverse events/side effects. Any discrepancies in data extraction were resolved through discussion or consultation with a third reviewer.

### Quality assessment

The included studies’ methodological quality and risk of bias were evaluated using the Cochrane Risk of Bias Tool: RCTs and ROBINS-I (Risk of Bias in Non-randomized Studies of Interventions) tool for observational studies. Two reviewers performed. The quality assessment was conducted independently, and disagreements were resolved through discussion or involving a third reviewer.

### Data synthesis

We examined the effectiveness of hydroxyurea in preventing secondary stroke in children with sickle cell anaemia by pooling relevant outcome data from the selected studies. The results of this systematic review were reported following the PRISMA guidelines for systematic reviews. A narrative synthesis of the findings was provided. No amendments were made to the protocol after its registration with PROSPERO.

## Results

### Included studies

A total of 43 studies were identified through the comprehensive search of electronic databases. After a thorough screening process, six studies met the pre-defined inclusion criteria and were included in this systematic review. These studies encompassed a variety of study designs, including RCTs, retrospective reviews, case series, and prospective cohort studies, providing a diverse range of evidence to address our research question. Among the six included studies, two were classified as high-quality: Ware and colleagues, a randomized control trial involving 35 children with sickle cell anaemia, and Abdullahi and colleagues, another randomized control trial with a substantial sample of 101 participants. Three studies fell into the moderate-quality category: Greenway and colleagues, a retrospective study; Lagunju and colleagues, another retrospective study; and Abdullahi and colleagues, a randomized control trial. The remaining study, Sumoza and colleagues, was categorised as low-quality due to its nature as a case series involving only five children, lacking a control group, and presenting limitations in sample size and design.

### Characteristics of included studies

Table 1 summarises the key characteristics of the included studies, including study design, number of participants, age range, hydroxyurea dosage, treatment duration, presence of a control group, main findings, and adverse events or side effects.

**Table 1:** Characteristics of included studies

References	Study design	No. participants	Age range	Hydroxyurea dosage	Treatment duration	Control group	Main findings	Adverse events/side effects
Ware *et al.* ^11^	Randomized control trial	35 children with SCA	Not specified	26.7±4.8 mg/kg per day	42±30 months	Yes (discontinued transfusions)	Stroke recurrence rate: 5.7 events per 100 patient-years - Overlapping hydroxyurea therapy reduced stroke recurrence to 3.6 events per 100 patient-years	Mild neutropenia (3.9±2.3×10^9^/l)
Greenway *et al.* ^12^	Retrospective study	35 patients with SCA	Not specified	Not specified	Median of 5.6 years	Not applicable	Recurrent stroke occurred in 29% of patients after switching to hydroxyurea.	Not specified
Sumoza *et al.* ^13^	Case series	5 children with SCA	Not specified	30 mg/kg/d (1 patient), 40 mg/kg/d (4 patients)	42–112 months	Not applicable	Hydroxyurea appeared to prevent recurrent stroke without major toxicity.	None reported
Abdullahi *et al.* ^14^	Randomised controlled trial	101 children with SCA	1–16 years	Fixed low-dose (10 mg/kg/day) or fixed moderate-dose (20 mg/kg/day)	Median follow-up of 1.6 years	Yes (low-dose versus moderate-dose)	No significant difference in stroke recurrence rates between low-dose and moderate-dose hydroxyurea groups. A total of 6 recurrent strokes and 2 deaths versus 5 recurrent strokes and 3 deaths occurred in the low- and moderate-dose groups, respectively.	No participants had hydroxyurea therapy stopped due to myelosuppression.
Lagunju *et al.* ^15^	Retrospective study	32 children with SCA	Not specified	Maximum dose ranged from 20-25 mg/kg/day	Not specified	Yes (declined HU therapy)	Secondary stroke incidence was significantly lower in the HU group. HU-treated children had better educational and motor outcomes.	Not specified
Abdullahi *et al.* ^16^	Randomised Control Trial	29	Not specified	20 mg/kg/day	Median 1.04 years	Not applicable	Eight children had a recurrent stroke, six of whom were prescribed hydroxyurea 20 mg/kg/day by 2 months after initial stroke. Six of the recurrent strokes occurred within 6 months of the initial stroke, two before hydroxyurea prescription. The stroke recurrence rate was 17.4 events per 100 patient-years	2 deaths

HU, hydroxyurea; SCA, sickle cell anaemia.

## Main findings

### Effectiveness of hydroxyurea in preventing recurrent strokes

The effectiveness of hydroxyurea in preventing recurrent strokes in children diagnosed with sickle cell anaemia was evaluated across the studies. In the study by Ware and colleagues, hydroxyurea, administered at a dosage of 26.7±4.8 mg/kg per day for an average treatment duration of 42±30 months, effectively prevented secondary strokes^11^. Although an initial stroke incidence rate of 0.88 per 100 patient-years was reported, six children experienced stroke recurrence, amounting to 17.4 events per 100 patient-years. Additionally, it resolved transfusional iron overload, indicating its potential to address related complications. Greenway *et al.* ^12^‘s retrospective review (2011) with a median treatment duration of 5.6 years found that 29% of patients experienced recurrent strokes after switching to hydroxyurea, indicating a potential limitation in preventing stroke recurrence. In contrast, Sumoza *et al.* ^13^ presented promising results in their case series involving five children. Hydroxyurea, administered at 30 mg/kg/d to 40 mg/kg/d for 42–112 months, effectively prevented recurrent strokes without major toxicity. Abdullahi *et al.* ^14^‘s randomised controlled trial 2023 with 101 children showed no significant difference in stroke recurrence rates between the low and moderate hydroxyurea dosage groups. This suggests that both low-dose (10 mg/kg/day) and moderate-dose (20 mg/kg/day) regimens may be equally effective in preventing recurrent strokes. Similarly, in Lagunju *et al.* ^15^‘s retrospective comparison study in 2013, hydroxyurea-treated children demonstrated a significantly lower incidence of secondary strokes They exhibited improved educational and motor outcomes despite a lack of specific treatment duration information. In Abdullahi *et al.* ^16^‘s 2019 study following a standard care protocol with 29 children, the hydroxyurea dose was 20 mg/kg/day, and the mean treatment duration was 1.25 years.

### Adverse effects of hydroxyurea

In examining the effectiveness of hydroxyurea as a treatment for children with SCA, it is essential to consider its safety profile. Adverse effects were assessed across the included studies, providing valuable insights into the tolerability of this therapy in paediatric patients. In the study by Ware *et al.* ^11^, which involved a prospective discontinuation approach, mild neutropenia (3.9±2.3×10^9^/l) was reported as an adverse event. Greenway *et al.* ^12^, in their retrospective review, did not record any adverse events related to hydroxyurea. However, their findings indicated that 29% of patients experienced recurrent strokes after transitioning to hydroxyurea, highlighting the importance of further investigation into its safety profile. Apart from these two, the other studies did not document any adverse effect connected with hydroxyurea. This observation suggests that the therapy prevented recurrent strokes in most cases without introducing major toxicity concerns.

## Discussion

The effectiveness of hydroxyurea in preventing recurrent strokes in children with SCA is a pivotal aspect of our systematic review. The results from various studies reveal a noteworthy variability in the response to hydroxyurea regarding preventing recurrent strokes. However, it is important to acknowledge that not all studies provided unequivocal support for hydroxyurea as a preventive measure. Greenway *et al.* ^12^ reported a significant portion of their patients experiencing recurrent strokes after transitioning to hydroxyurea. This raises essential questions about the universal efficacy of this treatment approach. Furthermore, the study by Abdullahi *et al.* ^14^ indicated no significant difference in stroke recurrence rates between low and moderate hydroxyurea dosage groups. This suggests that the dose may not be the sole determinant of effectiveness, calling for a more nuanced understanding of dosing strategies.

The variable duration of hydroxyurea treatment across the studies is another significant factor that warrants consideration. The diversity in treatment durations could potentially contribute to the observed outcome differences. Notably, some studies did not specify the treatment duration, making it challenging to draw firm conclusions about the optimal duration of therapy. Nevertheless, Lagunju *et al.* ^15^‘s retrospective comparison study is remarkable for its findings on improved educational and motor outcomes in hydroxyurea-treated children. This aspect highlights the potential holistic benefits of hydroxyurea treatment in children with SCA, transcending its role as a preventive measure for strokes.

In parallel, examining the safety profile of hydroxyurea in children with SCA is a paramount component of a comprehensive assessment of its utility. Ware *et al.* study reported mild neutropenia as an adverse event associated with hydroxyurea^11^. This underscores the imperative need for diligent monitoring of blood counts during treatment to ensure the safety of patients. However, it is essential to note that the presence or absence of adverse events related to hydroxyurea exhibited considerable variability across the studies. These observed adverse events, such as mild neutropenia, should be meticulously weighed against the potential benefits of hydroxyurea in preventing recurrent strokes and improving haematological parameters.

In light of these findings, it is evident that hydroxyurea can effectively prevent recurrent strokes in children with sickle cell anaemia. However, the complexity of the disease and the variable response among individuals highlight the need for a more tailored approach. The optimal dosage and treatment duration remain areas of ongoing investigation, demanding further research. Careful and continuous monitoring of haematological parameters during therapy ensures patient safety and treatment effectiveness.

Future research should prioritise refining dosing regimens, considering individual patient characteristics to personalise treatment approaches. Additionally, comprehensive studies should delve into the long-term effects of hydroxyurea treatment to provide a more holistic understanding of its impact on children with SCA. Furthermore, exploring potential ancillary benefits beyond stroke prevention, including educational and motor outcomes, will significantly enhance these young patients’ overall quality of life.

This systematic review evaluates the clinical evidence surrounding hydroxyurea’s role in preventing secondary strokes in paediatric SCA patients. It provides a broad perspective by including various study types, such as RCTs and observational studies. Furthermore, the study’s relevance to clinical practice is a notable strength, addressing a critical aspect of paediatric SCA management. Several limitations need to be acknowledged. The included studies exhibited significant heterogeneity, including variations in design, patient characteristics, dosages, and treatment durations. This heterogeneity makes direct comparisons challenging and may introduce bias into the analysis. Moreover, the study’s focus on peer-reviewed, English-language publications might introduce publication bias by excluding relevant non-English studies and unpublished data.

## Conclusion

This systematic review underscores the potential of hydroxyurea as a therapeutic avenue for preventing secondary strokes in paediatric SCA patients. While some studies reported positive outcomes, notably reducing stroke recurrence, others revealed variability in response and safety profiles. Hydroxyurea’s effectiveness and safety appear to vary among individuals, emphasising the need for tailored approaches. Optimal dosages and treatment durations remain areas of active investigation, highlighting the complexity of managing paediatric SCA patients. The observed adverse effects, primarily mild neutropenia, should be balanced against the potential benefits of stroke prevention and improved haematological parameters. Moreover, hydroxyurea’s potential ancillary benefits, including enhanced educational and motor outcomes, hold promise for improving the overall quality of life for children with SCA. This review underscores the importance of further research to refine dosing strategies, personalise treatment based on individual patient characteristics, assess long-term effects, and explore holistic benefits beyond stroke prevention. Such efforts will contribute to advancing the management of paediatric SCA, ultimately improving the well-being of affected children and their families.

## Ethical approval

None.

## Consent

None.

## Sources of funding

None.

## Author contribution

Conceptualization: N.A. Writing of first draft: all authors. Writing of final draft: all authors.

## Conflicts of interest disclosure

All authors declare no conflicts of interest.

## Research registration unique identifying number (UIN)

PROSPERO (york.ac.uk), CRD42023460588.

## Guarantor

Nicholas Aderinto.

## Data availability statement

No new datasets were generated for this review.

## Provenance and peer review

Not commissioned, externally peer-reviewed.
